# Supplementary material for: Chromothripsis during telomere crisis is independent of NHEJ, and consistent with a replicative origin
Source: Genome Res. 2019 May;29(5):737–49. doi: 10.1101/gr.240705.118 (PMC6499312; doi:10.1101/gr.240705.118)
Supplement: Supplemental Material [file supp_gr.240705.118_Supplemental_file_1.zip › contigs/annotated_contigs/DB51/contig.2.DB51_length_860_mean_cov_8.1511627907.docx]

**DB51_length_860_mean_cov_8.1511627907**

```
C|GATATGAACAGATACTTCACAAAAGAAGACATTCATGCTGCCAACAAACATATGAAAAAAAGCTCATTATCACTGGTCATTAGAGAA
  >chr10:31852070-31852538 - E=1e-266
ATGCAAAACAAAACCACAATGAGATACCATCTTACACCAGTTAGAATGGAAGTCATTAAAAAGTCAGGAAACAACAGATGCTGGGAGGA

TGTGCAGAAATAGGAACACTTCTTTTACACTGTTGGTGGGAATGTAAATTAGTTCAACCATTGTGGAAGATGGTGTGGTGATTCCTCAA

GGATGTAGAACCAGAAAATACCATTTGGCCCAGCAATCCCATTACTGGGTATATACCCAAAGGATTACAAATCATTCTGCTATAAAGAC

ACACGCACACATGTTTACTGAGGCACTATTCACAATAGCAAAGACTTGGAACCAATCCAAATGCCCATCAATGATAGACTGCATAAAGA

AAATGTGGCACATATACACCAT|GGA|TTTAACCTGAAAGTGGTTTATTCTTGTTATCCAATCGTAGTTTCACTCTTTCGAACATGGTA
                       >chr10:31159665-31160059 - E=4e-213
TGAAAGCTATTTTCTTAAGCTCTCTATTTTTTTCTTTTCCTCCTTGGCCATATCTCCCCTTTCCTCTCCATCTGGTGAAGGAACACTGA

TCTGGACAGCTGAGCTCTGGTCATTGTTACATGCTTCTTTTTAGGCTCTGTGATCTTGGCCAAATAAGTAAACCTCAGTCCCTTCATCT

TTCCAATGGAGATAGTAATTCTCGTCCTGTAACAGTCAAATGCAGTAGGTATGGTTGATTCTCATTATTTGTAGTATTTATATTCTAGA

AGGCTATTAGGAACTCTGAATTAGCAAATACTGAGCCATTTGTTCCTAGGCGATATTCAAGG
```
